# Racial and ethnic disparities and socioeconomic determinants of male breast cancer mortality in the United States

**DOI:** 10.1007/s10549-025-07851-y

**Published:** 2025-11-21

**Authors:** Jincong Q. Freeman, Kent Schechter, Long C. Nguyen, Olasubomi J. Omoleye, Jared H. Hara

**Affiliations:** 1 Department of Public Health Sciences, The University of Chicago, 5841 S Maryland Ave, MC2000, Chicago, IL USA; 2 Cancer Prevention and Control Research Program, UChicago Medicine Comprehensive Cancer Center, Chicago, IL USA; 3 Center for Health and the Social Sciences, The University of Chicago, Chicago, IL USA; 4 Ben May Department for Cancer Research, The University of Chicago, Chicago, IL USA; 5 Department of Medicine, The University of Chicago Medical Center, Chicago, IL USA; 6 Department of Radiation Oncology, The Queen’s Medical Center, Honolulu, HI USA; 7 The University of Hawaii Cancer Center, Honolulu, HI USA

**Keywords:** Male breast cancer, Racial/ethnic disparities, Socioeconomic determinants, Mortality, National Cancer Database

## Abstract

**Purpose:**

Male breast cancer (mBC) is rare and accounts for ~ 1% of all breast cancer cases in the United States (US), and mBC incidence has risen in recent years. This study sought to examine mBC mortality disparities across racial/ethnic groups and associated socioeconomic determinants at the national level.

**Methods:**

This retrospective study analyzed the 2010–2021 National Cancer Database. Patients were eligible if they were ≥ 18 years, were male sex, and had stage I-IV disease, with available data on receptor status. Overall survival (OS) was modeled using Cox regression adjusting for demographic, socioeconomic, and clinicopathologic factors.

**Results:**

Of 20,470 mBC (mean age, 66.2 [SD, 12.6] years), 2.5% were Asian or Pacific Islander, 13.8% Black, 4.0% Hispanic, and 78.2% White. After controlling for clinicopathologic characteristics, Black patients had worse OS than White patients (adjusted hazard ratio [AHR], 1.22; 95% CI, 1.12–1.32); however, when further adjusting for socioeconomic factors, this difference was no longer significant (AHR, 1.09; 95% CI, 0.99–1.21). Hispanic patients (AHR, 0.76; 95% CI, 0.62–0.94) had a lower mortality risk. OS varied across tumor stages and molecular subtypes. In the triple-negative mBC cohort, Asian or Pacific Islander patients had worse OS than White patients (AHR, 2.35; 95% CI, 1.21–4.55), warranting further investigation. Additionally, lower median household income, lack of health insurance, Medicaid/Medicare, and comorbidities were associated with a higher mortality risk.

**Conclusion:**

Our findings highlight elevated mortality risks of mBC among Black patients, among Asian or Pacific Islander patients with TNBC, and associations with household income and insurance status. Interventions addressing socioeconomic inequities that impact access to cancer care programs and services may help reduce racial/ethnic disparities and improve mBC survival outcomes.

**Supplementary Information:**

The online version contains supplementary material available at 10.1007/s10549-025-07851-y.

## Introduction

Male breast cancer (mBC) is rare, constituting approximately 1% of all breast cancer cases in the United States (US) with an estimated 2,800 new cases in 2025 [1]. Men who identify as Black or African American have higher rates of breast cancer than those who identify as White, Hispanic, and Asian or Other Pacific Islander​ [2]. Due to its rarity, data on mBC outcomes by race/ethnicity and socioeconomic determinants is limited. Additionally, due to comparatively limited evidence, mBC often gets treated following the guidelines created for female breast cancer. Hormone receptor (HR)-positive mBC – similar to estrogen receptor-positive, postmenopausal breast cancer in women – is treated with tamoxifen and/or chemotherapy. Some patients with triple-negative breast cancer (TNBC) may be eligible for pembrolizumab. mBC tumors with specific genetic mutations (e.g., human epidermal growth factor receptor 2 [HER2], PD-1, and *PIK3CA*) inform the use of targeted therapies or immune checkpoint inhibitors [3]. Evidence suggests that, although similar in some regards, mBC differs in its biological and clinical behavior compared to female breast cancer,[4] highlighting the need for more focused research to better understand its distinct characteristics and differences to improve male patients’ health outcomes​.

Although less common than female breast cancer, the incidence of mBC has risen in recent years,[5, 6] driven in part by known risk factors such as family history, aging, obesity, and high-penetrance genes (i.e., *BRCA1* and *BRCA2*) that can elevate the risk for mBC by up to 80-fold [7]. Previous studies also have documented that mBC patients have lower 3-year and 5-year survival rates than female breast cancer patients for all stages of disease [8–10]. Limited studies of racial and ethnic disparities in mBC mortality have indicated that Black men experience worse survival outcomes than White men,[11–13] mirroring trends seen among women with breast cancer in the US [14]. Social determinants have been shown to contribute to disparities in female breast cancer mortality, including socioeconomic status, access to health care and services, and facility type [6]. While race and ethnicity are closely tied to socioeconomic factors in the US, addressing these factors reduces racial/ethnic disparities in female breast cancer risk and health outcomes[15]. 

As previous studies in mBC have largely focused on differences between White patients and Black patients and the HR-positive/HER2-negative molecular subtype, little to less is known about mBC mortality disparities in other racial/ethnic groups and other molecular subtypes in real-world setting at the national level [11, 16, 17]. To address these gaps, we examined disparities in overall survival (OS) of mBC by race/ethnicity and social determinants using a large US clinical oncology registry. We hypothesized that racial/ethnic minority groups and low socioeconomic indicators were associated with worse OS in mBC.

## Methods

### Study design and data source

This was a retrospective study of prospectively collected data from mBC patients registered in the 2010–2021 National Cancer Database (NCDB), collected by the Commission on Cancer of the American College of Surgeons and the American Cancer Society [18]. The NCDB captures approximately 72% of new cancer diagnoses each year from more than 1,500 Commission on Cancer-accredited cancer centers in the US [19, 20]. Because NCDB de-identified data does not identify clinical facilities, providers, or patients, the University of Chicago Institutional Review Board exempted the study from review. In compliance with the NCDB’s Data Use Agreement and to protect patients’ confidentiality, we suppressed reporting of all individual cell counts < 10 in descriptive tables. The study followed the Strengthening the Reporting of Observational Studies in Epidemiology (STROBE) reporting guideline [21]. 

### Eligibility and sample selection

Sample selection of male patients diagnosed with invasive breast carcinoma was illustrated in Supplementary Fig. 1. Briefly, patients were eligible if they 1) were at least 18 years of age at diagnosis; 2) were assigned male sex at birth; 3) had tumors classified as stage I, II, III, or IV by the American Joint Committee on Cancer (AJCC) staging system; 4) were diagnosed between 2010 and 2021; and 5) included data on tumor molecular subtype.

### Measures

The main outcome of interest was OS, which was defined as the time from the initial breast cancer diagnosis to death from any cause or last patient contact. According to the NCDB, vital status was not available for patients diagnosed in 2021 because of limited time for follow-up. Thus, these patients were not included in our survival analysis. Median follow-up (in months) for the patient cohort was reported. Tumor stage was defined by the AJCC staging system and classified as stage I, II, III, or IV.[22] Molecular subtypes of breast tumors were categorized as HR-positive/HER2-negative, HR-positive/HER2-positive, HR-negative/HER2-positive, and TNBC. Due to small sample sizes, the HR-positive/HER2-positive and HR-negative/HER2-positive groups were combined into a HER-positive (or HER2-enriched) category for regression analyses.

Racial/ethnic groups comprised non-Hispanic Asian or Pacific Islander, non-Hispanic Black, Hispanic, non-Hispanic White, and Other. Other is a racial/ethnic group listed in the NCDB and represents patients who were classified as Other by local cancer registries. The NCDB does not specifically define race/ethnicity classified into Other. Additional demographic, area-level socioeconomic, and clinicopathologic characteristics included age at diagnosis, year of diagnosis, percent no high school degree quartile based on residential geographic area (≥ 17.6%, 10.9–17.5%, 6.3–10.8%, and < 6.3%), median household income quartile (< $40,227, $40,227-$50,353, $50,354-$63,332, and ≥ $63,333), type of health insurance (Medicaid, Medicare, other government, private, uninsured), rural–urban residence (metropolitan, rural, and urban), type of facility/cancer program, the Charlson-Deyo comorbidity score (0, 1, and ≥ 2), tumor histologic type (ductal, ductal and lobular, lobular, or other), and tumor grade (1—low, 2—intermediate, and 3—high).

### Statistical analysis

First, we described the study cohort using standard summary statistics. We used analysis of variance or the Kruskal–Wallis test to compare the distributions of continuous variables. Categorical variables were compared using Pearson’s Chi-square test. For survival analysis, the Kaplan–Meier method was used to create Kaplan–Meier curves, calculate the median survival time (in months), and estimate 3-year, 5-year, and 10-year rates of OS across racial/ethnic groups. There was a statistically significant interaction between race/ethnicity and molecular subtype (interaction *P* = 0.046). The statistical test for the interaction term of race/ethnicity and tumor stage was not significant (*P* = 0.167). We conducted stratified log-rank tests to determine statistical significance when comparing survival functions across racial/ethnic groups, overall and by molecular subtype. Cox proportional hazards regression models were fit to further assess racial/ethnic and socioeconomic disparities in OS. A stepwise model-building approach was employed. Specifically, model 1 included age at diagnosis and race/ethnicity; in addition, model 2 included histologic type, AJCC stage, tumor grade, and Charlson-Deyo comorbidity score; and, in addition, model 3 included percent no high school degree quartile, median household income quartile, type of insurance, rural–urban residence, and facility type. A similar approach was implemented in the stratified Cox regression when stratifying by molecular subtype. Adjusted hazard ratios (AHR) and 95% confidence intervals (95% CI) were calculated. All statistical tests were two-sided at the 0.05 level of significance. All statistical analyses were performed using Stata, version 18 (StataCorp LLC, College Station, TX). Forest plots were created using the *forestploter* package in R (R Foundation for Statistical Computing). Data were analyzed between July 2023 and October 2024.

## Results

### Patient characteristics

We identified a total of 20,470 mBC patients diagnosed between 2010 and 2021 (Table 1). The mean age at diagnosis was 66.2 years (SD, 12.6). Most (78.2%) patients self-identified as non-Hispanic White, followed by 13.8% as non-Hispanic Black, 4.0% as Hispanic, 2.5% as non-Hispanic Asian or Pacific Islander, and 1.6% as Other. Overall, 40.2% reported at a median household income quartile of ≥ $63,333; 37.3% had private insurance, 52.3% were on Medicare while 4.9% were on Medicaid; 91.4% were diagnosed with stage I-III; 83.6% were HR-positive/HER2-negative; and 54.1% had grade 2 tumors. Compared with White mBC patients, Asian or Pacific Islander, Black, or Hispanic patients were diagnosed at younger age, at higher percent no high school degree quartiles, at lower median household income quartiles (except for Asian or Pacific Islander), were more likely to be uninsured or on Medicaid (all *P* < 0.001). Black patients and Hispanic patients were more likely to be diagnosed with TNBC and grade 3 tumors compared to other racial/ethnic groups (Table 1).

**Table 1:** Distributions of sociodemographic and clinicopathologic characteristics of male patients with breast cancer, overall and by race/ethnicity

	Overall	White	Black	Asian or Pacific Islander	Hispanic	Other	
Characteristic	N = 20,470	n = 16,015	n = 2817	n = 503	n = 818	n = 317	
	No. (%)	No. (%)	No. (%)	No. (%)	No. (%)	No. (%)	*P* value ^a^
Age at diagnosis (years), mean (SD)	66.2 (12.6)	67.2 (12.3)	63.2 (12.6)	63.1 (13.4)	61.0 (13.9)	63.4 (13.3)	< 0.001
*Percent no high school degree quartiles*^*b*^							
≥ 17.6%	3093 (17.5)	1744 (12.6)	841 (34.9)	97 (22.0)	357 (49.6)	54 (18.8)	< 0.001
10.9–17.5%	4390 (24.8)	3272 (23.7)	824 (34.2)	75 (17.0)	150 (20.8)	69 (24.0)	
6.3–10.8%	5108 (28.9)	4294 (31.0)	504 (20.9)	109 (24.8)	140 (19.4)	61 (21.2)	
< 6.3%	5099 (28.8)	4522 (32.7)	241 (10.0)	159 (36.1)	73 (10.1)	104 (36.1)	
*Median household income quartiles *^*c*^							
< $40,227	2853 (16.2)	1642 (11.9)	944 (39.2)	40 (9.1)	191 (26.6)	36 (12.5)	< 0.001
$40,227–$50,353	3601 (20.4)	2836 (20.6)	490 (20.4)	53 (12.0)	163 (22.7)	59 (20.5)	
$50,354–$63,332	4110 (23.3)	3354 (24.3)	450 (18.7)	70 (15.9)	181 (25.2)	55 (19.1)	
≥ $63,333	7089 (40.2)	5967 (43.2)	523 (21.7)	277 (63.0)	184 (25.6)	138 (47.9)	
*Type of health insurance*							
Uninsured	407 (2.0)	228 (1.4)	95 (3.4)	13 (2.6)	62 (7.6)	9 (2.8)	< 0.001
Private/managed care	7637 (37.3)	5925 (37.0)	1008 (35.8)	238 (47.3)	328 (40.1)	138 (43.5)	
Medicaid	1011 (4.9)	542 (3.4)	298 (10.6)	55 (10.9)	97 (11.9)	19 (6.0)	
Medicare	10,702 (52.3)	8797 (54.9)	1293 (45.9)	189 (37.6)	291 (35.6)	132 (41.6)	
Other government/unknown	713 (3.5)	523 (3.3)	123 (4.4)	< 10 (< 2.0)	40 (4.9)	19 (6.0)	
*Rural–urban area*							
Metropolitan	17,335 (86.8)	13,267 (85.1)	2557 (92.3)	471 (96.3)	773 (95.8)	267 (88.4)	< 0.001
Urban	2360 (11.8)	2092 (13.4)	189 (6.8)	17 (3.5)	30 (3.7)	32 (10.6)	
Rural	271 (1.4)	239 (1.5)	24 (0.9)	< 10 (≤ 1.0)	< 10 (≤ 1.0)	< 10 (≤ 1.0)	
*Facility type/cancer program*							
Community	1775 (8.9)	1483 (9.4)	168 (6.2)	40 (8.4)	59 (7.8)	25 (8.2)	< 0.001
Comprehensive community	8171 (40.9)	6660 (42.4)	962 (35.5)	180 (37.7)	269 (35.4)	100 (32.7)	
Academic/research	5770 (28.9)	4176 (26.6)	1032 (38.1)	174 (36.4)	280 (36.8)	108 (35.3)	
Integrated network	4256 (21.3)	3397 (21.6)	550 (20.3)	84 (17.6)	152 (20.0)	73 (23.9)	
*Charlson-Deyo comorbidity score*							
0	15,346 (75.0)	12,103 (75.6)	1966 (69.8)	396 (78.7)	628 (76.8)	253 (79.8)	< 0.001
1	3364 (16.4)	2601 (16.2)	517 (18.4)	69 (13.7)	132 (16.1)	45 (14.2)	
≥ 2	1760 (8.6)	1311 (8.2)	334 (11.9)	38 (7.6)	58 (7.1)	19 (6.0)	
*Histologic type*							
Ductal	17,717 (86.6)	13,950 (87.1)	2365 (84.0)	429 (85.3)	706 (86.3)	267 (84.2)	< 0.001
Lobular	552 (2.7)	464 (2.9)	60 (2.1)	< 10(< 1.5)	12 (1.5)	< 10 (< 3.0)	
Ductal and lobular	387 (1.9)	307 (1.9)	38 (1.3)	11 (2.2)	19 (2.3)	12 (3.8)	
Other	1814 (8.9)	1294 (8.1)	354 (12.6)	56 (11.1)	81 (9.9)	29 (9.1)	
*AJCC stage group*							
I	9411 (46.0)	7588 (47.4)	1087 (38.6)	244 (48.5)	357 (43.6)	135 (42.6)	< 0.001
II	6564 (32.1)	5120 (32.0)	930 (33.0)	150 (29.8)	256 (31.3)	108 (34.1)	
III	2725 (13.3)	2052 (12.8)	447 (15.9)	60 (11.9)	124 (15.2)	42 (13.2)	
IV	1770 (8.6)	1255 (7.8)	353 (12.5)	49 (9.7)	81 (9.9)	32 (10.1)	
*Molecular subtype*							
HR +/HER2-	15,972 (83.6)	12,660 (84.4)	2055 (79.1)	391 (83.2)	618 (82.5)	248 (84.6)	< 0.001
HR +/HER2 +	2154 (11.3)	1660 (11.1)	346 (13.3)	48 (10.2)	77 (10.3)	23 (7.8)	
HR-/HER2 +	231 (1.2)	165 (1.1)	38 (1.5)	13 (2.8)	< 10 (< 1.5)	< 10 (< 2.5)	
TNBC	758 (4.0)	521 (3.5)	158 (6.1)	18 (3.8)	45 (6.0)	16 (5.5)	
*Tumor grade*							
1	2653 (14.4)	2161 (14.9)	304 (12.4)	70 (16.0)	93 (12.8)	25 (8.8)	< 0.001
2	9965 (54.1)	7865 (54.2)	1299 (52.8)	237 (54.1)	392 (54.0)	172 (60.6)	
3	5796 (31.5)	4481 (30.9)	856 (34.8)	131 (29.9)	241 (33.2)	87 (30.6)	
Median follow-up time in months (IQR)	52.8 (28.7, 84.6)	53.7 (29.3, 85.8)	47.4 (26.2, 78.1)	54.8 (30.0, 83.7)	52.5 (26.7, 80.1)	52.3 (27.3, 85.2)	< 0.001

No, number; SD, standard deviation; IQR, interquartile range; AJCC, American Joint Committee on Cancer; HR, hormone receptor; HER2, human epidermal growth factor receptor 2; TNBC, triple-negative breast cancer

^a^ *P* values were calculated using ANOVA or Kruskal–Wallis tests for continuous data and Pearson’s *X*^*2*^ tests for categorical data

^b^ Defined as education attainment for patient residence areas and measured by matching the zip code of the patient recorded at the time of diagnosis against files derived from the 2016 American Community Survey data

^c^ Based on the 2016 American Community Survey data, spanning years 2012–2016 and adjusted for 2016 inflation

### Racial/ethnic and socioeconomic disparities in all-cause mortality

**Fig. 1:**
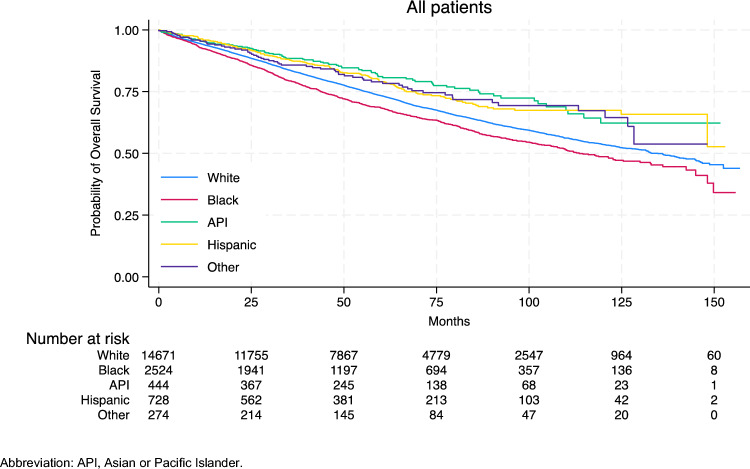
Kaplan–Meier curves for overall survival of male patients with breast cancer by race/ethnicity. *API*: Asian or pacific Islander

With a median follow-up of 52.8 months (interquartile range, 27.7–84.6), there were differences in OS curves between racial/ethnic groups overall (Fig. 1), with Black mBC patients having the shortest median survival time (113.0 months [95% CI, 106.7–130.0]; log-rank *P* < 0.001) (Table 2). Black patients experienced worse OS than other racial/ethnic patients in stage I, II, and III cohorts; the OS rate was similar by race/ethnicity for stage IV disease. When stratified by molecular subtype, we observed OS differences across racial/ethnic groups in the HR-positive/HER2-negative and TNBC cohorts (Supplementary Fig. 2). Black patients in the HR-positive/HER2-negative cohort and Asian or Pacific Islander patients in the TNBC cohort had the shortest median survival (Supplementary Table 1). Among all racial/ethnic groups, Black patients had the lowest 3-year, 5-year, and 10-year rates of OS overall (Table 2); however, these rates vary significantly across molecular subtypes, specifically among mBC patients diagnosed with HR-positive/HER2-negative (*P* < 0.001) and those with TNBC (*P* = 0.035) (Supplementary Table 1).

**Table 2:** Number of events and estimated probability of overall survival of male patients with breast cancer

Race/Ethnicity	No. of patients	No. of events	Median survival time in months (95% CI)	*P* value ^a^	3-Year survival, % (95% CI)	5-Year survival, % (95% CI)	10-Year survival, % (95% CI)
White	14,671	4526	134.8 (131.5, 140.2)	< 0.001	83.4 (82.7, 84.0)	73.4 (72.6, 74.2)	53.6 (52.3, 54.8)
Black	2524	860	113.0 (106.7, 130.0)		79.1 (77.3, 80.7)	68.4 (66.3, 70.3)	48.7 (45.5, 51.8)
API	444	89	NR		88.5 (84.9, 91.2)	81.1 (76.5, 84.9)	62.3 (52.9, 70.3)
Hispanic	728	156	NR		87.5 (84.7, 89.8)	79.0 (75.3, 82.2)	67.4 (62.4, 71.9)
Other	274	63	NR		85.7 (80.7, 89.6)	79.0 (72.8, 83.9)	67.3 (58.5, 74.7)

API, Asian or Pacific Islander; CI, confidence interval, NR, not reached

^a^ *P* values were calculated using the stratified log-rank test

In the adjusted Cox models (Table 3), mortality risk after adjusting for clinicopathologic characteristics (model 2) was significantly higher in Black mBC patients (AHR, 1.22; 95% CI, 1.12–1.32) and lower in Asian or Pacific Islander patients (AHR, 0.69; 95% CI, 0.54–0.88) compared to White patients. When further adjusting for socioeconomic factors (model 3), the OS difference was no longer significant between Black patients and White patients (AHR, 1.09; 95% CI, 0.99–1.21). Asian or Pacific Islander patients (AHR, 0.70; 95% CI, 0.54–0.90) and Hispanic patients (AHR, 0.76; 95% CI, 0.62–0.94) remained at a lower mortality risk (Table 3). Compared to mBC patients with a median household income of < $40,227, those of $40,227-$50,353 (AHR, 0.89; 95% CI, 0.80–0.99), $50,354-$63,332 (AHR, 0.85; 95% CI, 0.76–0.96), or ≥ $63,333 (AHR, 0.76; 95% CI, 0.67–0.86) had a lower risk of mortality. Patients with no insurance (AHR, 1.79; 95% CI, 1.42–2.26), Medicaid (AHR, 1.60; 95% CI, 1.35–1.89), or Medicare (AHR, 1.19; 95% CI, 1.09–1.30) had a higher mortality risk than those privately insured. Greater comorbidity scores were also associated with worse OS rates.

**Table 3:** Racial/ethnic differences in overall survival of male patients with breast cancer: Cox proportional hazards regression

	Model 1		Model 2		Model 3	
	AHR (95% CI)	*P* value	AHR (95% CI)	*P* value	AHR (95% CI)	*P* value
Age at diagnosis (per 10-year increase)	1.62 (1.59, 1.67)	< 0.001	1.71 (1.67, 1.76)	< 0.001	1.74 (1.67, 1.80)	< 0.001
*Race/Ethnicity*						
Black	1.44 (1.34, 1.55)	< 0.001	1.22 (1.12, 1.32)	< 0.001	1.09 (0.99, 1.21)	0.075
API	0.77 (0.62, 0.95)	0.013	0.69 (0.54, 0.88)	0.003	0.70 (0.54, 0.90)	0.006
Hispanic	0.94 (0.80, 1.10)	0.430	0.88 (0.74, 1.06)	0.183	0.76 (0.62, 0.94)	0.010
Other	0.83 (0.65, 1.06)	0.136	0.85 (0.65, 1.11)	0.234	0.86 (0.65, 1.15)	0.313
White	1.0 (reference)		1.0 (reference)		1.0 (reference)	
*Histologic type*						
Ductal			1.0 (reference)		1.0 (reference)	
Lobular			0.95 (0.78, 1.15)	0.595	0.94 (0.76, 1.15)	0.527
Ductal and lobular			0.88 (0.71, 1.08)	0.225	0.94 (0.75, 1.19)	0.629
Other			1.03 (0.92, 1.15)	0.568	1.01 (0.89, 1.14)	0.910
*AJCC stage group*						
I			1.0 (reference)		1.0 (reference)	
II			1.48 (1.38, 1.60)	< 0.001	1.46 (1.35, 1.58)	< 0.001
III			2.12 (1.95, 2.32)	< 0.001	2.07 (1.88, 2.28)	< 0.001
IV			7.69 (6.99, 8.45)	< 0.001	7.65 (6.89, 8.50)	< 0.001
*Molecular subtype*						
HR +/HER2-			0.55 (0.48, 0.64)	< 0.001	0.56 (0.48, 0.66)	< 0.001
HR +/HER2 +			0.62 (0.53, 0.72)	< 0.001	0.62 (0.52, 0.74)	< 0.001
HR-/HER2 +			0.52 (0.38, 0.72)	< 0.001	0.52 (0.36, 0.75)	0.001
TNBC			1.0 (reference)		1.0 (reference)	
*Tumor grade*						
1			1.0 (reference)		1.0 (reference)	
2			1.12 (1.02, 1.23)	0.027	1.10 (0.99, 1.23)	0.079
3			1.37 (1.24, 1.52)	< 0.001	1.32 (1.18, 1.48)	< 0.001
*Charlson-Deyo comorbidity score*						
0			1.0 (reference)		1.0 (reference)	
1			1.29 (1.20, 1.39)	< 0.001	1.29 (1.20, 1.40)	< 0.001
≥ 2			2.03 (1.86, 2.22)	< 0.001	2.04 (1.85, 2.24)	< 0.001
*Percent no high school degree quartiles*^*a*^						
≥ 17.6%					1.07 (0.74, 1.22)	0.277
10.9–17.5%					1.09 (0.98, 1.21)	0.118
6.3–10.8%					1.15 (1.05, 1.26)	0.003
< 6.3%					1.0 (reference)	
*Median household income quartiles *^*b*^						
< $40,227					1.0 (reference)	
$40,227–$50,353					0.89 (0.80, 0.99)	0.038
$50,354–$63,332					0.85 (0.76, 0.96)	0.006
≥ $63,333					0.76 (0.67, 0.86)	< 0.001
*Type of health insurance*						
Uninsured					1.79 (1.42, 2.26)	< 0.001
Private/managed care					1.0 (reference)	
Medicaid					1.60 (1.35, 1.89)	< 0.001
Medicare					1.19 (1.09, 1.30)	< 0.001
Other government/unknown					1.24 (1.02, 1.52)	0.033
*Rural–urban area*						
Metropolitan					1.0 (reference)	
Urban					1.05 (0.95, 1.16)	0.337
Rural					1.08 (0.82, 1.41)	0.593
*Facility type/cancer program*						
Community					1.0 (reference)	
Comprehensive community					1.10 (0.98, 1.24)	0.106
Academic/research					1.05 (0.92, 1.19)	0.486
Integrated network					1.07 (0.94, 1.22)	0.302

API, Asian or Pacific Islander; AHR, adjusted hazard ratio; CI, confidence interval; AJCC, American Joint Committee on Cancer; HR, hormone receptor; HER2, human epidermal growth factor receptor 2; TNBC, triple-negative breast cancer

^a^ Defined as education attainment for patient residence areas and measured by matching the zip code of the patient recorded at the time of diagnosis against files derived from the 2016 American Community Survey data

^b^ Based on the 2016 American Community Survey data, spanning years 2012–2016 and adjusted for 2016 inflation

When stratifying by molecular subtype (Supplementary Fig. 3), Black patients with HR-positive/HER2-negative mBC had a higher risk of mortality than White patients (AHR, 1.23; 95% CI, 1.12–1.35) (model 2); however, the difference between the two racial groups was no longer significant when further adjusting for socioeconomic characteristics (AHR, 1.09; 95% CI, 0.98–1.22) (model 3). Asian or Pacific Islander patients had a lower risk of mortality than White patients (AHR, 0.63; 95% CI, 0.48–0.82), and this difference persisted when additionally controlled for socioeconomic factors. For HER2-positive tumors, the OS rate was similar by race/ethnicity (Supplementary Fig. 3). In the TNBC cohort, Black patients experienced worse OS than White patients (AHR, 1.44; 95% CI, 1.08–1.91); however, this racial differences were not statistically significant after controlling for key clinicopathologic and socioeconomic covariates (AHR, 1.09; 95% CI, 0.74–1.61). Asian or Pacific Islander patients were at an increased mortality risk than White patients (AHR, 2.35; 95% CI, 1.21–4.55), after adjusting for clinicopathologic characteristics (Supplementary Fig. 3). Additionally, patients with a median household income of $50,354-$63,332 (AHR, 0.86; 95% CI, 0.76–0.98) or ≥ $63,333 (AHR, 0.73; 95% CI, 0.64–0.84) had a lower mortality risk than those of < $40,227. Compared to private insurance, no insurance (AHR, 1.68; 95% CI, 1.29–2.19), Medicare (AHR, 1.21; 95% CI, 1.10–1.33), or Medicaid (AHR, 1.73; 95% CI, 1.43–2.09) was associated with an increased mortality risk.

## Discussion

We compared the mortality of mBC in a large US cohort by racial/ethnic groups and socioeconomic factors. Mortality risk also varied across molecular subtypes. Black patients had a higher risk of mortality than White patients when adjusting for clinicopathologic factors. However, when further adjusting for socioeconomic indicators, the mortality risk between Black patients and White patients did not vary to a level of statistical significance. Overall, Asian or Pacific Islander and Hispanic patients had better OS than their White peers. In the TNBC cohort, Asian or Pacific Islander patients fared worse OS than White patients, meriting further investigation. Lower median household income quartiles, Medicaid, Medicare, or no insurance, and greater comorbidity scores were associated with a higher risk of mortality in mBC.

In this study, Black mBC patients consistently had higher all-cause mortality than White patients overall and across molecular subtypes, after adjusting for clinicopathologic characteristics. This racial disparity mirrors trends found in female breast cancer studies [14]. Notably, however, after further adjusting for area-level socioeconomic indicators, the survival difference between Black patients and White patients was no longer statistically significant. A recent comparative analysis of mBC has also documented a similar rate of OS between the two racial groups after controlling for both clinical and sociodemographic factors 23. Our findings suggest that alleviating socioeconomic inequities may reduce disparities in mBC mortality between Black patients and White patients.

We also found that after adjusting for clinicopathologic characteristics or further for socioeconomic factors, Asian or Pacific Islander and Hispanic patients with mBC displayed non-statistically significant trends toward slightly lower mortality risk compared with White patients, and this was consistent across molecular subtypes. However, it is worth noting that in the TNBC cohort, Asian or Pacific Islander patients experienced the lowest 5-year and 10-year rates of OS compared with other racial/ethnic categories. This is a surprising result, as previous research in TNBC have described Black men having significantly lower OS rates than White men, without much focus on other racial/ethnic groups displaying the same trend [24]. This result may be due to the small sample size for the Asian or Pacific Islander patient group. It is also possible that these survival disparities may be masked by aggregation of Asian and other Pacific Islander as a racial category [25]. Between 2010 and 2014, Asian women with TNBC had better survival of any racial demographic with stage I-III tumors but poorer survival in the metastatic setting [26]. Furthermore, Asian and other Pacific Islander women experienced the steepest increase of all racial/ethnic groups in breast cancer rates from 2012 to 2021, [14] combined with a significant increase in TNBC incidence between 2010 and 2019 among those ages ≥ 55 years [27]. This shift in TNBC incidence and lower survival rates in Asian or Pacific Islander women may contain parallels in male TNBC, which warrants future research.

Socioeconomic indicators correlated with mortality disparities in mBC, both overall and when stratified by molecular subtype. Across all mBC patients and within stage I-II and HR-positive/HER2-negative disease, higher median household income quartiles were associated with greater survival rates. An analysis of the 2004–2016 NCDB reported that higher income levels were associated with improved survival in mBC. However, this and other studies have not fully stratified patients based on molecular subtype and/or tumor stage [11, 12, 16, 23]. Having private health insurance was associated with a lower mortality risk compared to having no health insurance coverage, Medicaid, or Medicare. Patients with a higher median household income had better survival outcomes, aligning with findings from prior studies [6, 23, 28]. However, educational attainment, facility type, and rural–urban residence were not significantly or consistently associated with OS across molecular subtypes, though congruent with reports in mBC literature [11, 28]. Collectively, these findings suggest that differences in health insurance and household income may contribute to disparities in mBC mortality.

Several limitations of this study should be acknowledged. First, although the NCDB includes extensive clinicopathologic and certain relevant sociodemographic data, its mortality statistics only include all-cause mortality. It is worth examining disease-specific, progression-related, or other survival outcomes of mBC in future studies. The NCDB does not collect chemotherapy regimen-specific data, such as anthracyclines and taxane-based regimens. These systemic therapies may affect patients’ survival outcomes. Due to this limitation, we were unable to make conclusions about survival differences related to the type and intensity of treatment in mBC. Moreover, there are many unmeasured potential confounders not collected by the NCDB. Germline and somatic genetic mutations may influence associations between sociodemographic, clinicopathologic, and survival variables. Unmeasured socioeconomic variables – such as transportation barriers, environmental pollution, social support, lifestyle behavior, proximity to dedicated comprehensive cancer centers, and personal and family history – likely influence the survival differences across racial/ethnic groups observed in the current study. Therefore, investigators should consider these key variables in their future analyses. Lastly, given the nature of the NCDB registry and this retrospective study design, the generalizability of our findings may be limited. However, the racial demographics of mBC patients in the NCDB generally reflect the US population, and our findings are consistent with prior studies using the Surveillance, Epidemiology, and End Results data [12, 16]. 

In conclusion, Black patients with mBC had a greater risk of mortality than White patients when controlling for clinicopathologic features; however, the risk was similar between the two racial groups after further controlling for socioeconomic indicators. Compared with White mBC patients, Asian or Pacific Islander and Hispanic patients had better survival outcomes, except for Asian or Pacific Islander patients who had higher mortality from TNBC. Lower median household income quartiles, lack of health insurance coverage or public insurance, and greater comorbidity scores were associated with a higher risk of all-cause mortality. Our findings highlight racial/ethnic disparities and socioeconomic determinants of survival outcomes in mBC across molecular subtypes. Strategies to address socioeconomic inequities at the national level that impact access to comprehensive cancer care programs and services may help reduce racial/ethnic disparities and improve survival outcomes of the mBC population in the US.

## Supplementary Information

Below is the link to the electronic supplementary material.

Supplementary file1 (PDF 559 KB)

## Data Availability

Data for this study were obtained from the US National Cancer Database. Investigators affiliated with Commission on Cancer-accredited cancer programs can request the National Cancer Database Participant User File by submitting an application to the American College of Surgeons via https://www.facs.org/quality-programs/cancer-programs/national-cancer- database.
