# Supplementary material for: Racial and ethnic disparities and socioeconomic determinants of male breast cancer mortality in the United States
Source: Breast Cancer Res Treat. 2025 Nov 21;215(1):9. doi: 10.1007/s10549-025-07851-y (PMC12638332; doi:10.1007/s10549-025-07851-y)
Supplement: Supplementary file 1 — Supplementary file1 (PDF 559 KB) [file 10549_2025_7851_MOESM1_ESM.pdf]

Supplemental Materials

Supplementary Figure 1. CONSORT Diagram of Cohort Selection of Male Patients with Breast Cancer in the National Cancer Database

Supplementary Table 1. Number of Events and Estimated Probability of Overall Survival among Male Patients with Breast Cancer, Stratified by Molecular Subtype

Supplementary Figure 2. Kaplan-Meier Curves for Overall Survival of Male Breast Cancer across Racial/Ethnic Groups, Stratified by Subtype

Supplementary Figure 3. Racial/Ethnic Differences in Overall Survival of Male Breast Cancer, Stratified by Molecular Subtype: Cox Proportional Hazards Regression

Supplementary Figure 1. CONSORT Diagram of Cohort Selection of Male Patients with Breast Cancer in the National Cancer Database

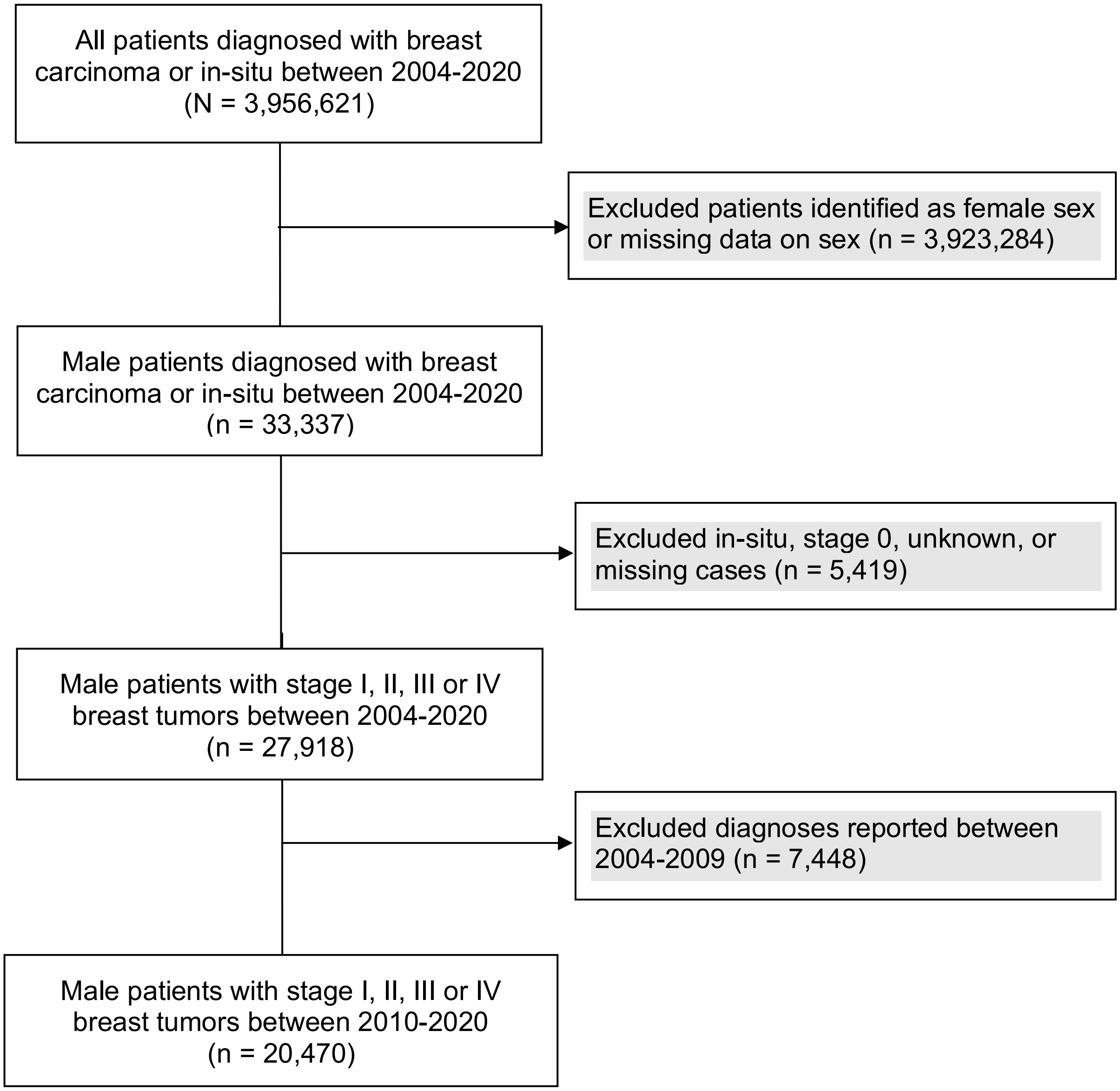

Supplementary Table 1. Number of Events and Estimated Probability of Overall Survival among Male Patients with Breast Cancer, Stratified by Molecular Subtype

Molecular subtype	Race/Ethnicity	No. of subjects	No. of events	Median survival time in months (95% CI)	*P* value [a]	3-Year survival, % (95% CI)	5-Year survival, % (95% CI)	10-Year survival, % (95% CI)
**HR+/HER2-**								
	White	11534	3370	136.8 (132.1, 145.9)	<0.001	85.3 (84.6, 85.9)	75.0 (74.1, 75.9)	54.3 (52.9, 55.7)
	Black	1816	579	122.7 (110.4, 145.0)		81.0 (79.1, 82.9)	70.1 (67.6, 72.4)	51.2 (47.5, 54.9)
	API	346	64	NR		90.8 (87.1, 93.6)	82.7 (77.5, 86.8)	62.1 (51.0, 71.4)
	Hispanic	549	107	148.1 (148.1, -)		89.1 (86.0, 91.6)	81.3 (77.1, 84.8)	69.3 (63.4, 74.4)
	Other	213	45	NR		87.1 (81.5, 91.1)	82.8 (76.3, 87.7)	69.9 (59.5, 78.2)
**HR+/HER2+**								
	White	1527	498	122.1 (114.5, -)	0.413	81.3 (79.2, 83.2)	70.8 (68.2, 73.2)	51.0 (46.8, 55.2)
	Black	313	105	109.8 (85.1, -)		80.3 (75.3, 84.5)	71.2 (65.2, 76.3)	44.0 (34.1, 53.4)
	API	41	<10	NR		86.4 (70.3, 94.1)	86.4 (70.3, 94.1)	69.9 (41.3, 86.5)
	Hispanic	67	18	NR		84.8 (72.7, 91.8)	73.0 (58.2, 83.3)	58.9 (40.7, 73.2)
	Other	20	<10	NR		77.4 (49.8, 91.0)	61.0 (31.4, 80.9)	50.8 (21.6, 74.1)
**HR-/HER2+**								
	White	147	43	NR	0.123	76.3 (68.2, 82.5)	72.5 (64.0, 79.4)	62.7 (51.7, 71.9)
	Black	36	14	NR		68.4 (48.9, 81.8)	57.4 (37.7, 72.9)	53.6 (34.1, 69.7)
	API	10	<10	NR		——	——	——
	Hispanic	<10	<10	NR		——	83.3 (27.3, 97.5)	——
	Other	<10	<10	NR		——	——	——
**TNBC**								
	White	488	182	NR	0.035	67.7 (63.2, 71.8)	62.0 (57.2, 66.5)	56.4 (50.9, 61.5)
	Black	148	65	63.7 (43.7, -)		64.0 (55.3, 71.5)	52.3 (43.0, 60.8)	47.3 (37.7, 56.3)
	API	18	11	43.7 (24.6, -)		54.6 (29.2, 74.2)	28.3 (6.5, 55.7)	28.3 (6.5, 55.7)
	Hispanic	39	<10	NR		86.8 (71.2, 94.3)	76.4 (57.9, 87.6)	76.4 (57.9, 87.6)
	Other	14	<10	NR		83.3 (48.2, 95.6)	60.8 (25.0, 83.6)	——

Abbreviations: API, Asian or Pacific Islander; HR, hormone receptor; HER2, human epidermal growth factor receptor 2; TNBC, triple-negative breast cancer; CI, confidence interval, NR, not reached.
[a] *P* values were calculated using the stratified log-rank test.

Supplementary Figure 2. Kaplan-Meier Curves for Overall Survival of Male Breast Cancer across Racial/Ethnic Groups, Stratified by Subtype

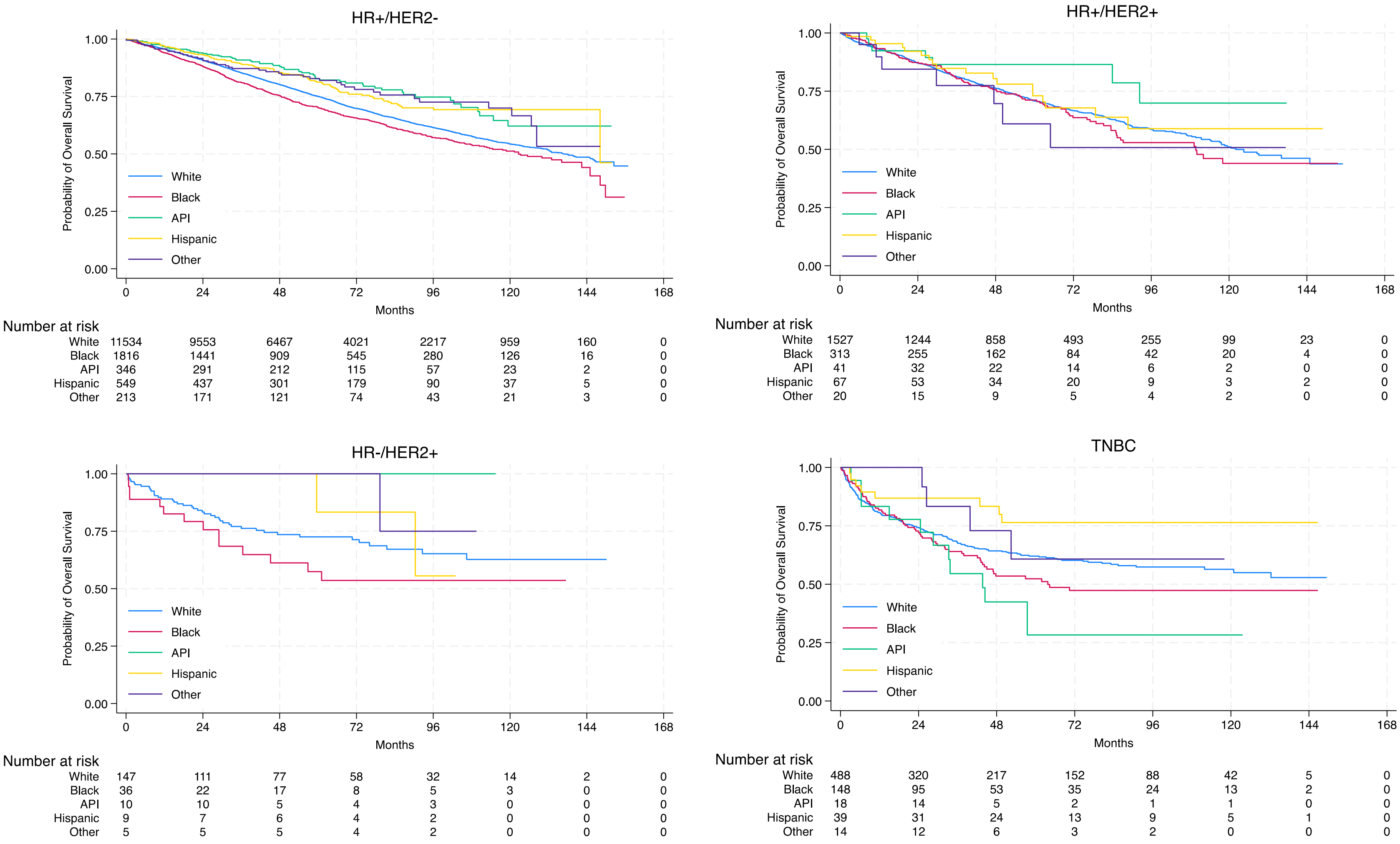

Abbreviations: API, Asian or Pacific Islander; HR, hormone receptor; HER2, human epidermal growth factor receptor 2; TNBC, triple-negative breast cancer.

Supplementary Figure 3. Racial/Ethnic Differences in Overall Survival of Male Breast Cancer, Stratified by Molecular Subtype: Cox Proportional Hazards Regression

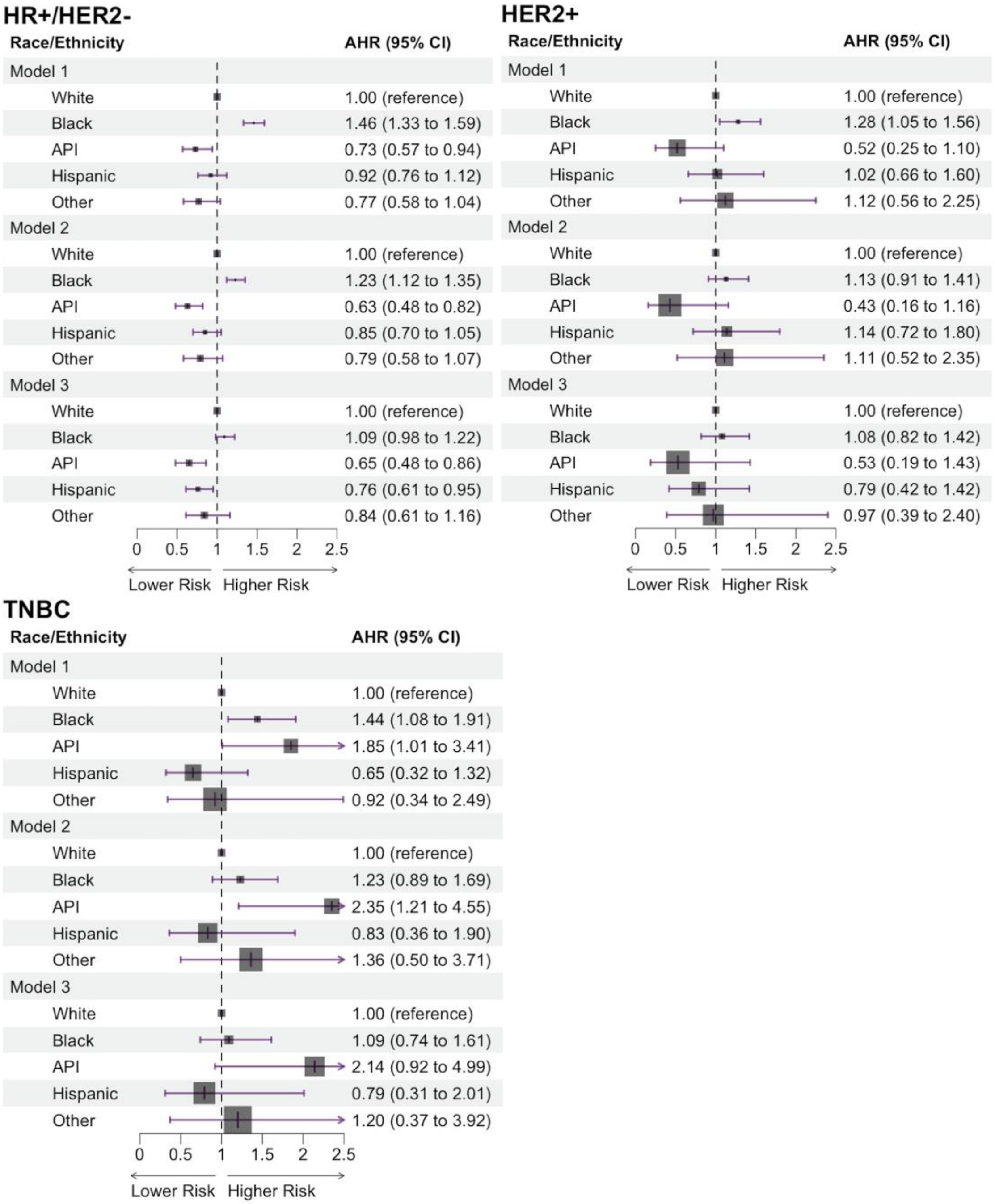

Abbreviations: API, Asian or Pacific Islander; AHR, adjusted hazard ratio; CI, confidence interval; HR, hormone receptor; HER2, human epidermal growth factor receptor 2; TNBC, triple-negative breast cancer.

Model 1 included age at diagnosis only. **Model 2** included age at diagnosis, histologic type, AJCC stage group, tumor grade, and Charlson-Deyo comorbidity score. **Model 3** included age at diagnosis, histologic type, AJCC stage group, tumor grade, Charlson-Deyo comorbidity score, percent no high school degree quartile, median household income quartile, type of health insurance, rural-urban area, and facility type.
